# Two-Dimensional Analysis of Smooth Pursuit Eye Movements Reveals Quantitative Deficits in Precision and Accuracy

**DOI:** 10.1167/tvst.8.5.7

**Published:** 2019-09-11

**Authors:** Lee Mcilreavy, Tom C. A. Freeman, Jonathan T. Erichsen

**Affiliations:** 1 School of Optometry and Vision Sciences, Cardiff University, Cardiff, UK; 2 School of Psychology, Cardiff University, Cardiff, UK

**Keywords:** pursuit gain, smooth pursuit, eye movements, gaze holding, velocity

## Abstract

**Purpose:**

Small moving targets are followed by pursuit eye movements, with success ubiquitously defined by gain. Gain quantifies accuracy, rather than precision, and only for eye movements along the target trajectory. Analogous to previous studies of fixation, we analyzed pursuit performance in two dimensions as a function of target direction, velocity, and amplitude. As a subsidiary experiment, we compared pursuit performance against that of fixation.

**Methods:**

Eye position was recorded from 15 observers during pursuit. The target was a 0.4° dot that moved across a large screen at 8°/s or 16°/s, either horizontally or vertically, through peak-to-peak amplitudes of 8°, 16°, or 32°. Two-dimensional eye velocity was expressed relative to the target, and a bivariate probability density function computed to obtain accuracy and precision. As a comparison, identical metrics were derived from fixation data.

**Results:**

For all target directions, eye velocity was less precise along the target trajectory. Eye velocities orthogonal to the target trajectory were more accurate during vertical pursuit than horizontal. Pursuit accuracy and precision along and orthogonal to the target trajectory decreased at the higher target velocity. Accuracy along the target trajectory decreased with smaller target amplitudes.

**Conclusions:**

Orthogonal to the target trajectory, pursuit was inaccurate and imprecise. Compared to fixation, pursuit was less precise and less accurate even when following the stimulus that gave the best performance.

**Translational Relevance:**

This analytical approach may help the detection of subtle deficits in slow phase eye movements that could be used as biomarkers for disease progression and/or treatment.

## Introduction

Smooth pursuit eye movements allow us to keep the retinal image of small moving targets on our foveae. Success at pursuit tasks traditionally has been defined by pursuit gain, the ratio of eye velocity to target velocity. Pursuit gain is a measure of accuracy that often is taken to represent an individual's overall pursuit performance. It has been used widely in a variety of clinical studies to determine the degree to which this type of eye movement has been spared in disease, including research on multiple sclerosis,^1^,^2^ schizophrenia,^3^,^4^ Parkinson's disease,^5^ Huntington's disease,^6^ Alzheimer's disease,^7^ Crouzon syndrome,^8^ and autism.^9^ However, as others have argued,^10^,^11^ the use of pursuit gain ignores any changes in the variability of smooth pursuit eye velocity; that is, the precision with which observers tracked the target. Concentrating on accuracy alone also could be misleading: in theory, overall pursuit accuracy could remain unchanged across multiple trials for a given observer, despite each trial containing eye movements that differ in their velocity distribution.

The use of pursuit gain in the literature also is one-dimensional – it only relates to the accuracy of the eye movement parallel to the target's trajectory. A tacit assumption, therefore, is that eye movements are not occurring orthogonal to the target's trajectory. This contrasts with the analysis of oculomotor behavior in other areas, such as sustained fixation, with the evidence suggesting that eye motion is two-dimensional (2D), displacing along horizontal and vertical axes.^12^,^13^ Pursuit eye movements are likely to be no exception. For instance, in one of the few studies of the 2D nature of smooth pursuit, Shanidze et al.^14^ sampled eye position as observers tracked a target that moved through the eight cardinal directions. The subsequent analyses quantified the proximity of eye position samples (60 Hz) to the pursuit target, and the results demonstrated that, for all pursuit directions, eye position varied not just along the target trajectory, but also orthogonal to it.

We used a similar 2D analysis to Shanidze et al. to investigate accuracy and precision of eye movements as a function of target direction, velocity, and amplitude. However, our analysis was built on samples of eye velocity, not position, which allows a more direct comparison with classic measures of pursuit gain where appropriate. The choice to use velocity also reflects the fact that pursuit is primarily driven by motion,^15^ though there is increasing evidence of a positional input as well.^16^

The 2D approach taken here also parallels recent analysis of stationary fixation by Cherici et al.^13^ In their study, the mean precision of ocular drift velocity during fixation in naïve observers was reported to be less than 56 arcmin/s^13^ whereas during pursuit eye velocity along the target trajectory typically is less precise.^11^ On this basis, we would firstly predict that precision should be lower along the target trajectory than orthogonal to it. Cherici et al.^13^ also described 2D velocity samples that show that ocular drift velocity during fixation is generally along the vertical axis with a downward bias, although other idiosyncratic drift patterns occurred. In other words, when the target is stationary (i.e., has zero velocity), vertical eye velocity is more variable (i.e., less precise) than horizontal. This leads to our second prediction that eye velocity orthogonal to the target trajectory should be more precise during vertical than horizontal pursuit. In contrast to the predictions for relative direction, we finally predicted that neither target velocity nor target amplitude should have any effect on eye velocity orthogonal to target trajectory, since the velocity of the target orthogonal to its trajectory will always be zero.

## Methods

### Participants

This study was done in accordance with the tenets of the Declaration of Helsinki. Informed consent was obtained from all observers after they received an explanation of the nature and possible consequences of the study. Ethical approval was granted by the Research Ethics and Audit Committee of the School of Optometry and Vision Sciences, Cardiff University. Fifteen observers (mean age, 29.47 ± 7.90 years; range, 21–49 years; nine males) participated, and all had either normal or corrected-to-normal vision.

### Materials

Stimuli were presented using a CRT projector (Multiscan VPH 1272QM; Sony, Tokyo, Japan) onto the rear of a large screen (200 × 155 cm) positioned 140 cm from the observer in a completely darkened room. The projector was modified so that only the central green cathode ray tube was used. All stimuli were generated using the OpenGL graphics library driven by the Delphi programming environment (Version 7; Borland Software Company; Cupertino, CA) and displayed using a GeForce 7300 LE graphics card (NVIDIA; Santa Clara, CA) at a frame rate of 72 Hz with a resolution of 1024 × 768 pixels. Observers (with their head supported with chin and head rests) viewed the stimuli binocularly, while eye position was recorded monocularly using a tower-mounted EyeLink 1000 (SR-Research, Ottawa, Canada). Observers wore their habitual refractive correction (if any), and the eye with better acuity was used for recording. In the case of equal acuities, the right eye was calibrated by default. The manufacturer reports that the accuracy and precision of the system are 0.5° and 0.01°, respectively.^17^

### Stimuli and Procedure

The pursuit target was a 0.4° green dot (1.24 cd/m^2^) that moved against a black background at either 8°/s or 16°/s and with a peak-to-peak amplitude of either 8°, 16°, and 32°. Pursuit was along two directions, horizontal and vertical, with each direction run as a separate block and repeated. The order of the experimental blocks was counterbalanced across observers. Before each experimental block, eye movements were calibrated using a nine-point grid with an interstimulus spacing of 16°. The EyeLink 1000 software evaluated each calibration and was repeated if necessary. Observers were instructed to follow the target to the best of their ability. The experiment was self-paced, requiring observers to initiate each trial with a single button press on a wireless keyboard. For each pursuit trial, the target stepped by half of the peak-to-peak amplitude opposite to the subsequent initial pursuit direction and remained stationary for 2 seconds before continuously moving left and right, or up and down, for 24 seconds. The initial 2 seconds where the target remained stationary was chosen as we wished to run this experimental protocol on other observers with visual impairment, some of whom may take longer to move their eyes to the peripheral location.^18^ For purposes of comparison, observers also fixated a 0.4° target presented straight ahead, for 26s.

### Analysis

Eye movement data were analyzed offline using scripts written in MATLAB (Version 2014b; MathWorks, Natick, MA). The initial 2 seconds of the pursuit data, when the target was stationary, were discarded. Similarly, the initial 2 seconds of fixation data also were discarded. Eye position data from the central 70% of each sweep of the target were used in the subsequent analysis to avoid pursuit initiation artefacts and anticipatory slowing of eye movements associated with the reversal of target direction. Eye movement data for repeated trials were concatenated before they were parsed into their respective pursuit directions (right, left, up, down). The initial 2 seconds of fixation data also were discarded and the entire 24 seconds of remaining fixation data used for comparison with pursuit data.

Eye position data were filtered using a fourth order Butterworth filter with a 60 Hz cutoff, before temporal differentiation to obtain velocity, acceleration, and jerk. Artefacts, representing blinks and sporadic dropped data, were identified as those regions where jerk exceeded an arbitrary threshold of 3 × 10^6^ °/s^3^. Saccades were identified in the remaining data and removed using a velocity criterion (mean eye velocity plus a multiple of the standard deviation [SD]) that was unique to each observer and adjusted by the experimenter to ensure accurate identification of saccades.

**Figure 1:**
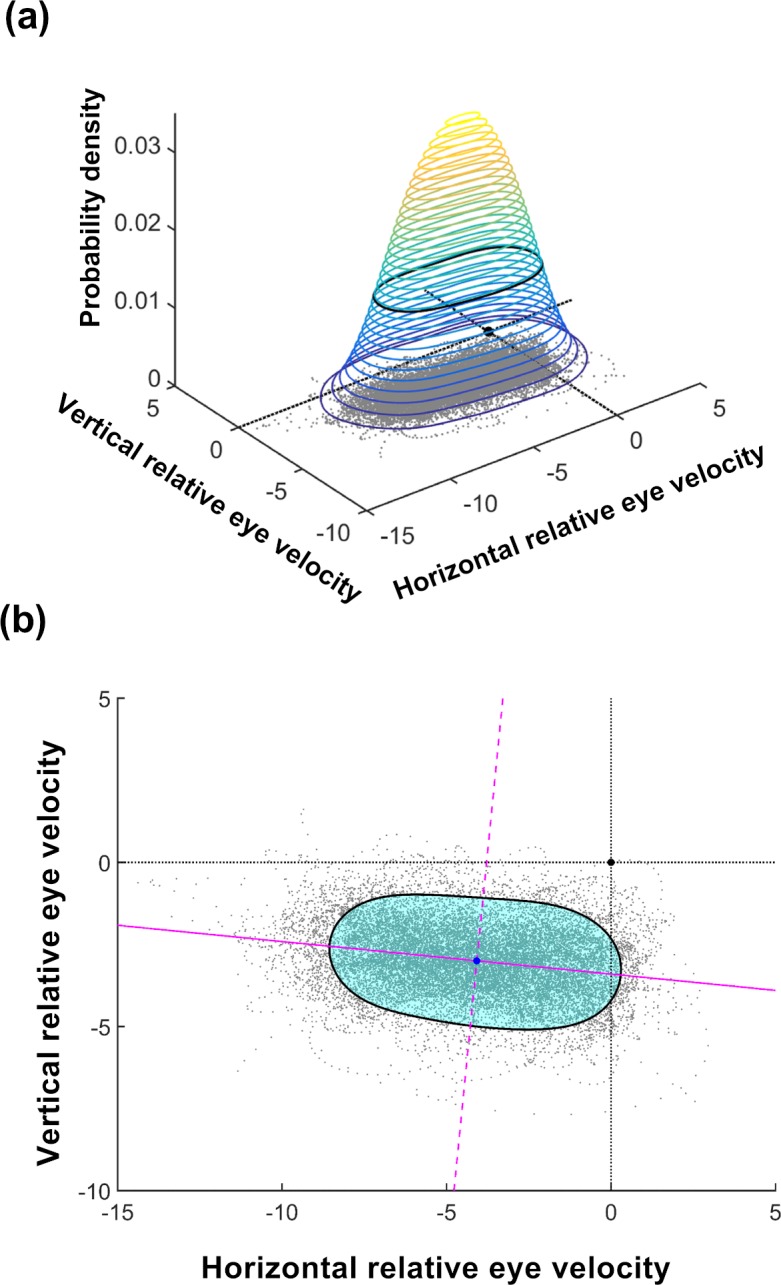
(a) A hypothetical distribution of eye velocity error during a pursuit trial. Each data point (*gray*) represents a raw eye velocity sample minus the target velocity. Hence, the origin of the distribution (*black dot*) is equal to the target velocity, and the proximity of the distribution to the origin is a measure of pursuit accuracy, while the spread of the distribution is a measure of pursuit precision. Superimposed on this hypothetical distribution is the corresponding bPDF, shown as a series of color-coded isocontours. Each contour defines an area of equal probability density, with warmer colors indicating a higher probability density. The isocontour that encompasses 68% of the highest probability densities of eye velocities is depicted in *black*. (b) A 2D view of the same 68% isocontour superimposed on the hypothetical velocity error distribution. The area bounded by the isocontour (*cyan*) is a measure of overall pursuit precision. Because the spread of eye velocities may not be equal along all directions, the isocontour may not be symmetric. The *solid* and *dashed magenta lines* indicate the major (most eye velocity spread, lowest precision) and minor (least eye velocity spread, greatest precision) axes of the isocontour, respectively. Taking the ratio of minor to major axis lengths provides an index of precision asymmetry (shape factor), while the orientation of the major axis with respect to the *x*-axis indicates the direction of greatest imprecision with respect to the target trajectory (major versus orthogonal). The precision along the individual minor and major axes can be calculated from the total area bounded by the isocontour and the shape factor. The proximity of the isocontour centroid (*blue dot*) to the origin of the distribution, using the magnitude of the centroid *x*- and *y*-coordinates, measures pursuit accuracy.

For each pursuit trial, a distribution of velocity errors was obtained from the difference between each remaining eye velocity sample and the target velocity. A bivariate probability density function (bPDF) then was computed for this distribution using an open source script,^19^ with a 256 × 256 mesh and a 1° kernel bandwidth.^20^ The isocontour that encompassed 68% of the highest probability density data was subjected to further analysis to determine the accuracy and precision along and orthogonal to the target trajectory (Fig. 1). An isocontour area of 68% is analogous to ±1 SD of a univariate normal distribution,^21^ and follows the convention in similar analytical approaches used to study fixation stability.^12^,^22^

Eye velocity accuracy was quantified using the coordinates of the isocontour centroid. Recall that these are all differences expressed relative to the target velocity at the origin, so smaller values reflect greater accuracy. Thus, during horizontal pursuit, the *x*-coordinate of the centroid represents accuracy along the target trajectory, whereas the *y*-coordinate represents accuracy orthogonal to target trajectory. As the origin of the velocity distribution is equal to the target velocity, the smaller the magnitude of the *x* or *y* coordinate, the more accurate the eye velocity during pursuit along or orthogonal, respectively, to the target trajectory.

To determine whether the minor or major axis of the isocontour lay along the target trajectory, we calculated the angle between the major axis of the isocontour and the *x*-axis. Since these data were axial, it was possible for measurements to be diametrically opposed (e.g., 0° vs. 180°) yet still have a common (i.e., horizontal) axis. Therefore, rather than taking the arithmetic mean of these major axis orientations (i.e., 90°, which would imply a vertical orientation), we doubled all major axis angles (e.g., 0° vs. 360°) before undertaking circular statistical analyses^23^ to eliminate this potential ambiguity. Once completed, all major axis orientations then were reported as the back-transformed, unambiguous axial data.

While the total area enclosed by the isocontour is a measure of the total precision of eye velocity, we also wished to know if eye velocities were distributed nonuniformly (i.e., an asymmetric isocontour). For this reason, we calculated the shape factor for the isocontour as the ratio of the minor to major axis. However, a ratio can obscure the actual values of the degree of precision. Thus, eye velocity precision along the individual minor and major axes (i.e., the velocity range about the centroid) was calculated from the ratio of the major and minor axes (shape factor) and the total area enclosed by the isocontour.

### Statistics

Circular statistics for angular data were computed using an open source toolbox for MATLAB.^24^,^25^ A Kuiper's v test was used to determine statistically whether angular data were distributed uniformly around a circle (null hypothesis) or had an a priori specified mean direction (alternative hypothesis). A repeated measures analysis of variance (ANOVA) was used to analyze each performance metric with respect to target direction, velocity, and amplitude. Mauchly's test for sphericity was performed along with each ANOVA, and when the assumption of sphericity had been violated, the degrees of freedom were adjusted using a Greenhouse-Geisser correction. A Bonferroni correction for multiple comparisons was applied to all post hoc tests. All ANOVAs were performed using JASP (version 0.8.2; JASP Team). Error bars represent the 95% confidence intervals and were calculated using the Cousineau-Morey method.^26^,^27^

## Results

**Figure 2:**
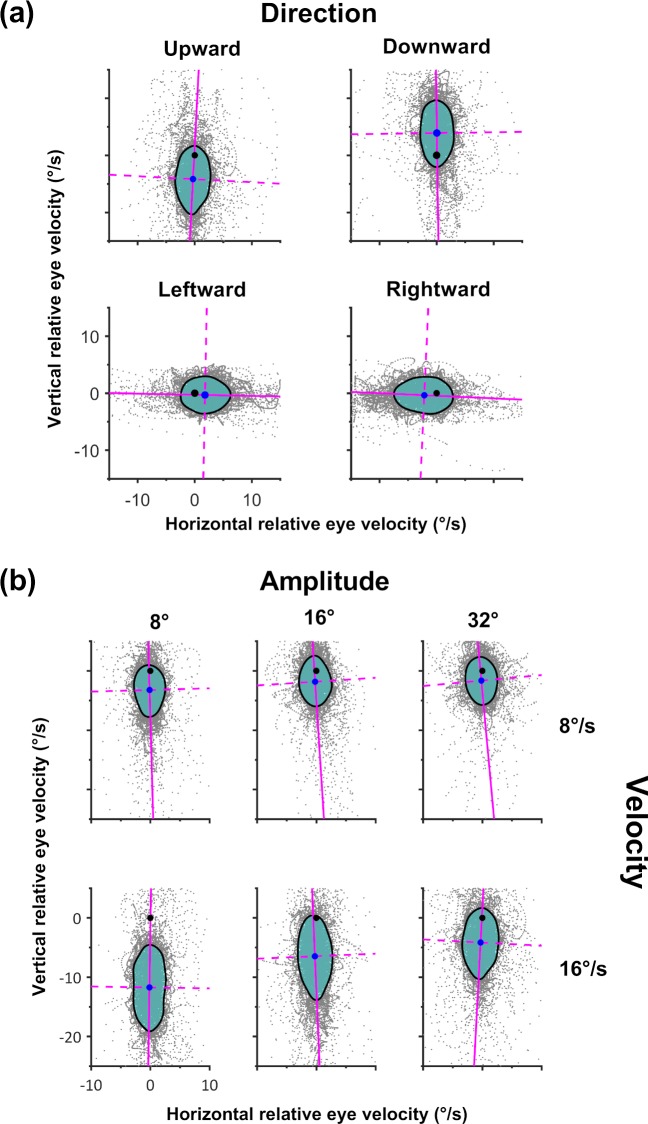
Typical results for the main effect of target direction, velocity, and amplitude (data taken from participant 15). The format of each isocontour Figure is the same as Figure 1, with *gray* data indicating the distributions of target-relative eye velocity. The 68% isocontour is represented by the *cyan* area bounded by a *black* outline. (a) The major axis of each isocontour (*solid magenta line*) is aligned along the target trajectory (velocity 16°/s; amplitude 32°). Vertical pursuit directions had lower shape factors and lower accuracy. Note the direction of inaccuracy is such that the isocontours are located behind the target with respect to its trajectory, indicating pursuit gain was less than 1. (b) Each column depicts the effect of target velocity and each row depicts the effect of target amplitude (target direction upward). Higher target velocity reduced accuracy and precision along the target trajectory, with lower shape factors. When pursuit amplitude decreased, shape factor decreased and the accuracy and precision along the target trajectory were lower.

A summary of the pursuit performance for each of the target manipulations is shown in Figure 2. In Figure 2a, the major axis of each isocontour (solid magenta line) is aligned along the target trajectory (in this case velocity 16°/s; amplitude 32°) for all directions of pursuit. However, for vertical pursuit directions, the isocontours were more spread (i.e., less precision) along the target trajectory (i.e., lower shape factors) and had less accuracy. Note that the mean relative velocity is such that the isocontours always were located behind the target with respect to its trajectory, indicating pursuit gain was less than 1.

Figure 2b depicts the effects of target velocity and target amplitude (for an upward target direction) on pursuit. Higher target velocity reduced accuracy and resulted in more spread along the target trajectory (i.e., lower shape factors). When pursuit amplitude decreased, shape factor also was lower, and the accuracy and precision along the target trajectory decreased.

### Angular Data

Before examining the results of the repeated measures ANOVA, we analyzed the orientations of the major axes of the velocity isocontours as a function of target direction. We had predicted that eye velocity would be more variable along the target trajectory than orthogonal to it. If this prediction were correct, then the major axes of the velocity isocontours should be aligned horizontally during horizontal pursuit and aligned vertically during vertical pursuit.

**Figure 3:**
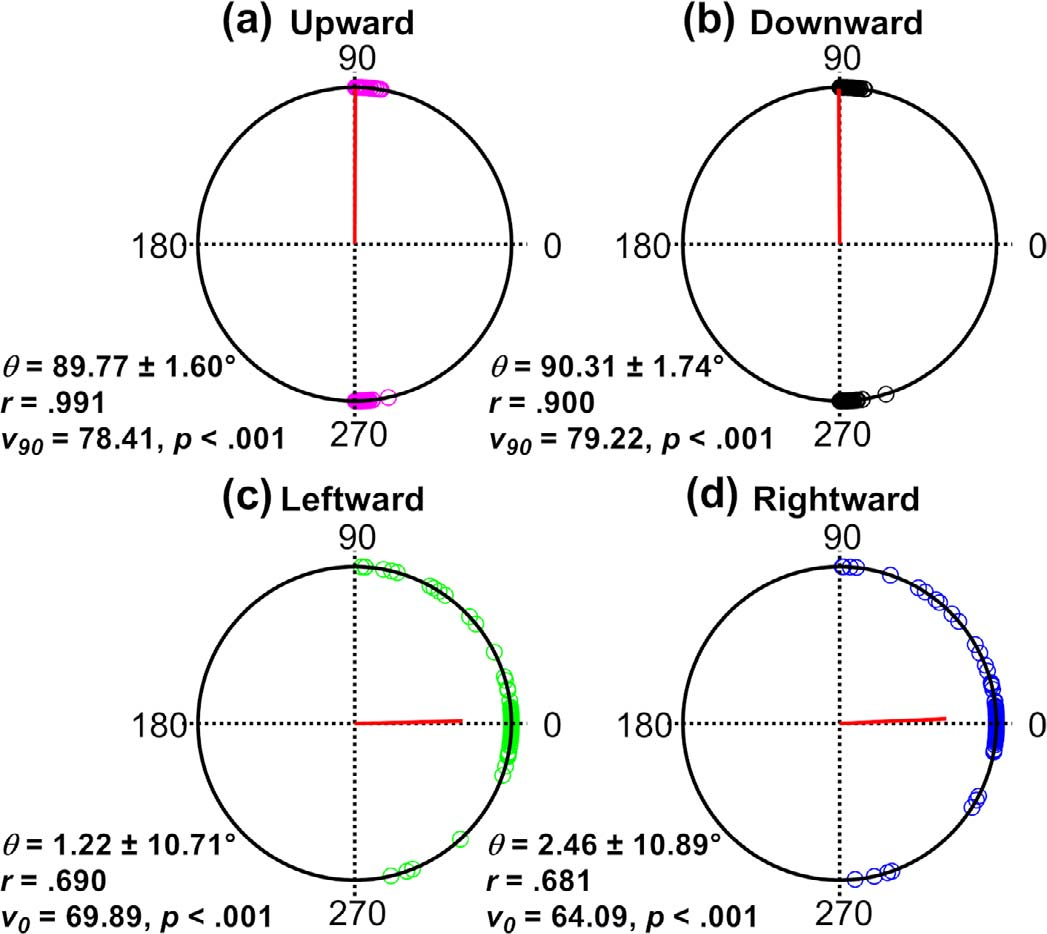
Polar distributions of the major axis orientation of the each isocontour calculated with respect to the *x*-axis. Each unfilled *circle point* on the unit circle represents a single trial, and each plot contains all trials for all observers for a given pursuit direction. The magnitude (*r*) and orientation (*θ*) of the resultant vector (*red line*) are given for each pursuit direction. The magnitude of the resultant vector is a measure of circular spread. The closer the magnitude is to 1 (i.e., the closer the *red line* is to the unit circle), the more concentrated the data. Kuiper's v test result (*v_α_*) indicates whether the distribution had significant mean direction (α). For all pursuit directions, the major axis was aligned, hence eye velocity was most imprecise, along rather than orthogonal to the target trajectory. The magnitude of the resultant vector was larger for vertical pursuit directions, indicating less circular spread of data. Indeed, the 95% confidence intervals for vertical pursuit were almost an order of magnitude smaller than for horizontal pursuit.

Figure 3 depicts the polar distributions of the angle formed between the major axis of the isocontour and the *x*-axis for during pursuit along each direction. The orientation and magnitude of the mean resultant vector (*r*, red line extending from the center of each plot) indicates the mean orientation of the major axes and the extent to which the data are circularly spread (i.e., variability in the orientation of the major axes); the closer this resultant vector is to the edge of the unit circle (i.e., *r* = 1), the more concentrated the underlying distribution is with respect to its orientation.

Figures 3a and 3b illustrate the results for vertical pursuit directions, upward and downward. In both cases, the resultant vector is aligned along the 90° to 270° axis and almost extends to the unit circle (*r* ≥ 0.90), indicating that underlying distribution was highly concentrated at this orientation (i.e., the orientations of major axes were parallel to the target trajectory). In addition, the upward and downward data were tested to determine whether they had a mean orientation along the 90° to 270° axis (i.e., Kuiper's v test with an a priori mean direction of 90°) with both results highly significant (*P* < 0.001). Essentially the same results were obtained for horizontal directions, leftward and rightward (Figs. 3c, 3d). As with vertical pursuit directions, the major axes for pursuit of a horizontal target were significantly oriented along the 0° to 180° axis (*P* < 0.001). However, the value of the resultant vectors was somewhat lower (*r* = 0.69 and 0.68, respectively).

There was no significant difference in the angular data between rightward or leftward pursuit directions (*P* = 0.551), or between upward and downward (*P* = 0.551). Taken together, the results in Figure 3 showed that the major axes had significant orientations of 0° (i.e., horizontal) and 90° (i.e., vertical) for horizontal and vertical pursuit, respectively. In terms of eye velocity distribution, the major axis denotes greater variability. Hence, for horizontal and vertical pursuit, there was less precision along the respective target direction than orthogonal to it, consistent with our first prediction. Given these findings, eye velocities along the major and minor axes will be referred to as eye velocities along and orthogonal to the target trajectory, respectively.

We noted that the magnitude of the resultant vector (*r*) was smaller for horizontal pursuit directions, suggesting greater variation in the major axis orientation data across observers on these trials. This may reflect changes to the distribution of pursuit precision along and orthogonal to the target trajectory, which may be apparent in our other analyses (i.e., shape factor; see below).

### Repeated Measures ANOVAs

The results of the repeated measures ANOVA for target direction, velocity, and amplitude on each of the pursuit metrics are presented in Table 1, and are discussed in turn.

**Table 1:** ANOVA Results for the Three Dependent Measures (Accuracy, Precision, and Shape Factor)

	*df*	*F*	*P*	*η*^2^
Accuracy_A_				
Direction	2.01, 28.16	24.24	<0.001	0.634
Velocity	1.00, 14.00	204.86	<0.001	0.936
Amplitude	2.00, 28.00	187.51	<0.001	0.931
D × V	3.00, 42.00	32.02	<0.001	0.696
D × A	3.32, 46.52	2.90	0.040	0.172
V × A	2.00, 28.00	180.28	<0.001	0.928
D × V × A	3.11, 43.58	0.96	0.424	0.064
Accuracy_O_				
Direction	3.00, 42.00	1.20	0.320	0.079
Velocity	1.00, 14.00	23.67	<0.001	0.628
Amplitude	2.00, 28.00	0.15	0.858	0.011
D × V	3.00, 42.00	0.97	0.416	0.065
D × A	2.56, 35.87	1.61	0.210	0.103
V × A	1.28, 17.95	1.66	0.219	0.106
D × V × A	2.89, 40.44	1.43	0.248	0.093
Precision_A_				
Direction	1.17, 16.38	0.43	0.551	0.030
Velocity	1.00, 14.00	70.01	<0.001	0.833
Amplitude	1.21, 16.95	11.40	0.002	0.449
D × V	1.84, 25.76	0.96	0.391	0.064
D × A	1.84, 25.71	9.38	0.001	0.401
V × A	2.00, 28.00	3.07	0.067	0.180
D × V × A	2.79, 39.11	8.37	<0.001	0.374
Precision_O_				
Direction	1.04, 14.50	4.07	0.061	0.225
Velocity	1.00, 14.00	9.83	0.007	0.412
Amplitude	1.16, 16.29	0.24	0.667	0.017
D × V	1.12, 15.66	3.83	0.065	0.215
D × A	1.92, 26.88	0.11	0.894	0.007
V × A	1.39, 19.51	3.32	0.072	0.192
D × V × A	2.16, 30.18	0.45	0.659	0.031
Shape factor				
Direction	1.07, 14.95	18.79	<0.001	0.573
Velocity	1.00, 14.00	39.93	<0.001	0.740
Amplitude	1.49, 20.83	28.31	<0.001	0.669
D × V	1.24, 17.39	0.25	0.858	0.018
D × A	2.64, 36.89	9.92	<0.001	0.415
V × A	2.00, 28.00	7.02	0.003	0.334
D × V × A	2.63, 36.84	7.31	<0.001	0.343

Subscript letters A and O denote measures taken along and orthogonal to the target trajectory, respectively.

#### Effect of Target Direction

**Figure 4:**
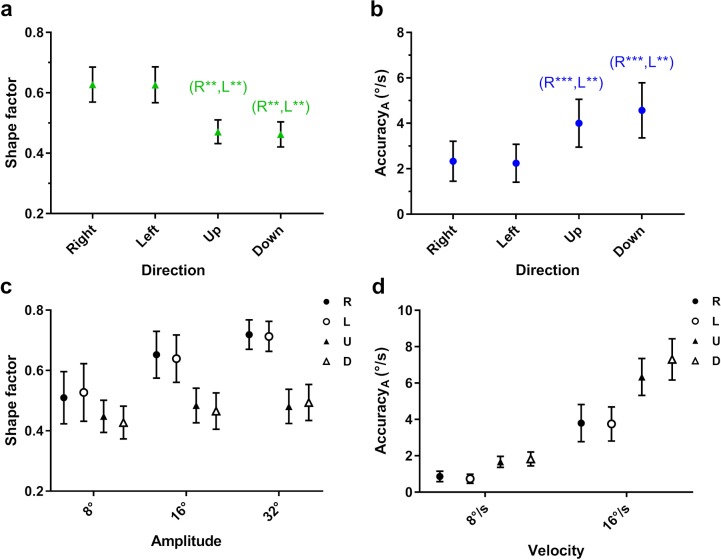
Mean (a) shape factor and (b) accuracy along the target trajectory as a function of target direction. (c) Mean shape factor as a function of velocity by target direction, and (d) mean accuracy along the target trajectory as a function of velocity by target direction. All *error bars* indicate the Cousineau-Morey 95% confidence intervals. The significance of post hoc comparisons is displayed above the data. ‘R' and ‘L' indicate those results that were significantly different from rightward and leftward target directions, respectively. The levels of statistical significance are denoted as: ***P < 0.001; **P < 0.010; *P < 0.050.

All post hoc analyses of significant main effects with direction (i.e., shape factor and accuracy along the target trajectory) indicated no significant differences between the two horizontal pursuit directions or between vertical pursuit directions (Figs. 4a, 4b).

Vertical pursuit precision was distributed significantly more asymmetrically (i.e., lower shape factors) than horizontal pursuit precision (Fig. 4a). This finding would appear to agree with our second prediction, that eye velocity orthogonal to the target trajectory should be more precise during vertical than horizontal pursuit. However, there were no main effects for precision along (*P* = 0.551) or orthogonal (*P* = 0.061) to the target trajectory with direction. In other words, the lower shape factor during vertical pursuit may have arisen due to changes in the precision along both planes, the result of which could be exaggerated by the ratio of the two.

The significant interaction effect of direction *x* amplitude for shape factor (*P* < 0.001) indicated that shape factor for vertical pursuit was always lower (i.e., more asymmetric) than that for horizontal pursuit across all three target amplitudes (Fig. 4c). Moreover, manipulating target amplitude had no effect on shape factors for vertical pursuit, whereas those for horizontal pursuit decreased with target amplitude. In other words, during horizontal pursuit, eye velocity is more variable, and its distribution shows a greater alignment with the axis of the target trajectory with increased amplitude.

Pursuit accuracy along the target trajectory was significantly lower for vertical than for horizontal pursuit (Fig. 4b). The significant interaction effect of direction *x* velocity indicated that accuracy was lower for vertical than horizontal pursuit across both velocities (Fig. 4d). Furthermore, accuracy was lower for all pursuit directions at the higher velocity, where the difference in accuracy between vertical and horizontal pursuits became even larger.

#### Effect of Target Velocity

**Figure 5:**
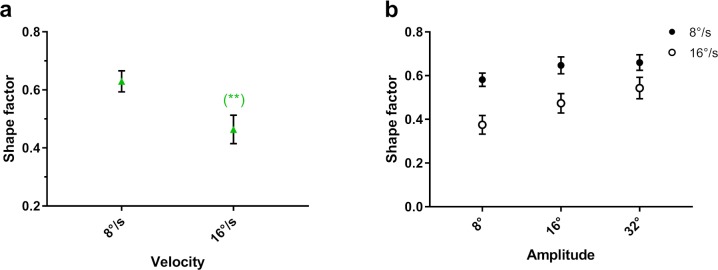
Mean shape factor as a function of (a) target velocity and (b) target amplitude by velocity. The significance of post hoc comparisons is displayed above the data. The format and depiction of the levels of statistical significance are the same as in Figure 4.

The higher target velocity had lower shape factors; that is, the distributions were more asymmetric, elongated along the axis of the target trajectory (Fig. 5a). A significant interaction effect of velocity *x* amplitude indicated that the higher target velocity had lower shape factors across all three target amplitudes. In addition, as target amplitude decreased, the shape factors for both velocities decreased, while the difference between them increased (Fig. 5b).

**Figure 6:**
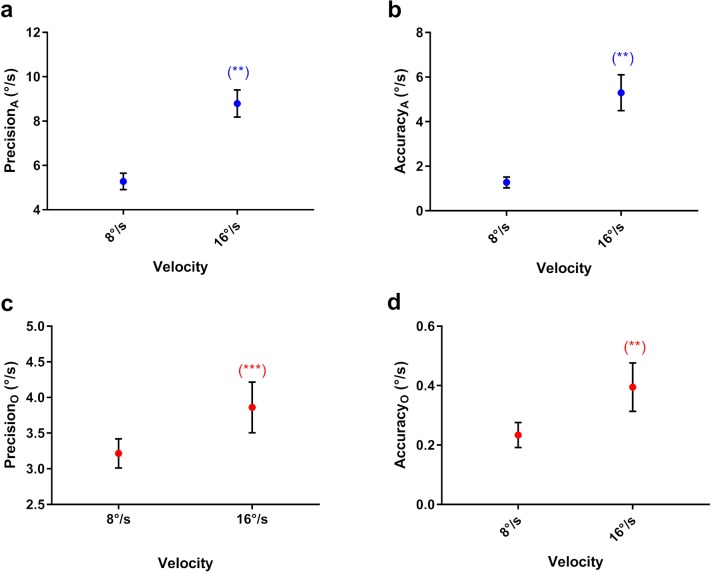
Mean (a) precision and (b) accuracy along the target trajectory. Mean (c) precision and (d) accuracy orthogonal to the target trajectory. The significance of post hoc comparisons is displayed above the data. The format and depiction of the levels of statistical significance are the same as in Figure 4.

Finally, the precision and accuracy of pursuit along and orthogonal to the target trajectory decreased at the higher target velocity for horizontal and vertical target movements (Figs. 6a–d). This significant impact of target velocity contradicted the first part of our third prediction.

#### Effect of Target Amplitude

**Figure 7:**
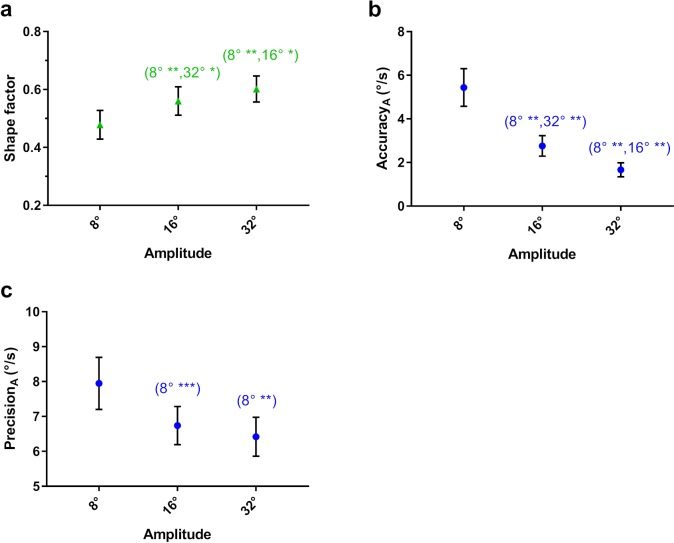
Mean (a) shape factor and (b) accuracy along the target as a function of target amplitude. Mean (c) precision along the target trajectory as a function of target amplitude. The significance of post hoc comparisons is displayed above the data. The format and depiction of the levels of statistical significance are the same as in Figure 4. Note that ‘8°', ‘16°', and ‘32°' indicate those results that were significantly different from 8°, 16°, and 32° target amplitudes, respectively.

Similar to the effect of higher target velocity, when pursuit amplitude decreased, shape factor decreased (Fig. 7a). Post hoc results showed a significant decrease in precision along the target trajectory (major axis) when pursuit amplitude decreased, but no significant differences orthogonal to the target trajectory (minor axis; Fig. 7c), suggesting that the changes in shape factor were driven by the major axis and confirms our initial expectation that target amplitude does not influence eye velocity orthogonal to the trajectory of the target. In addition to these results, the accuracy of pursuit along the target trajectory decreased with smaller target amplitudes (Fig. 7b).

### Saccades

When following a moving target, if the observer is unable to move their eye with the appropriate velocity, the resulting position and velocity error may be reduced by means of a saccade.^28^ Thus, the quality of pursuit is inversely related to the number of saccades. We analyzed the number of saccades initiated by each participant on each trial. We only considered the central 70% of each sweep to avoid those saccades associated with the target reversal.

#### Saccade Number

**Figure 8:**
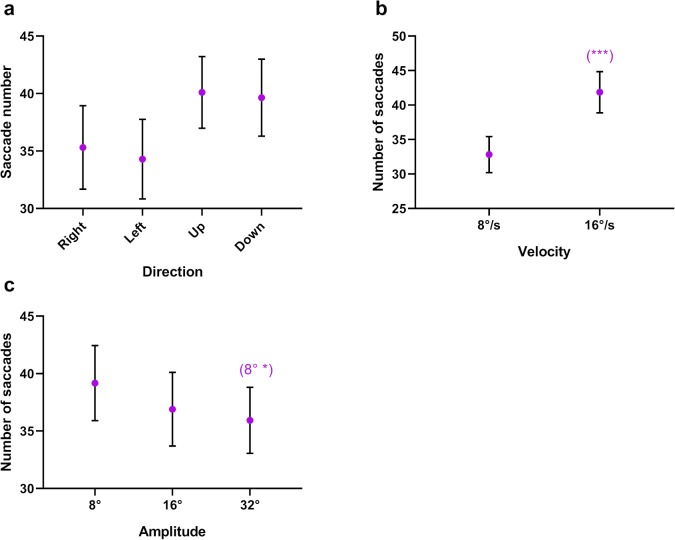
Mean number of saccades as a function of target (a) direction, (b) velocity, and (c) amplitude. The significance of post hoc comparisons is displayed above the data. The format and depiction of the levels of statistical significance are the same as in Figure 4.

There was no significant main effect of direction (*F*_1.19,16.66_ = 2.973, *P* < 0.001, *η*^2^ = 0.175) on the number of saccades (Fig. 8a). However, there was a significant main effect of velocity (*F*_1,14_ = 60.316, *P* < 0.001, *η*^2^ = 0.812) and amplitude (*F*_2,28_ = 5.748, *P* = 0.008, *η*^2^ = 0.291) on the number of saccades, with more saccades initiated at the higher target velocity (Fig. 8b) and at the smallest target amplitude (Fig. 8c).

**Figure 9:**
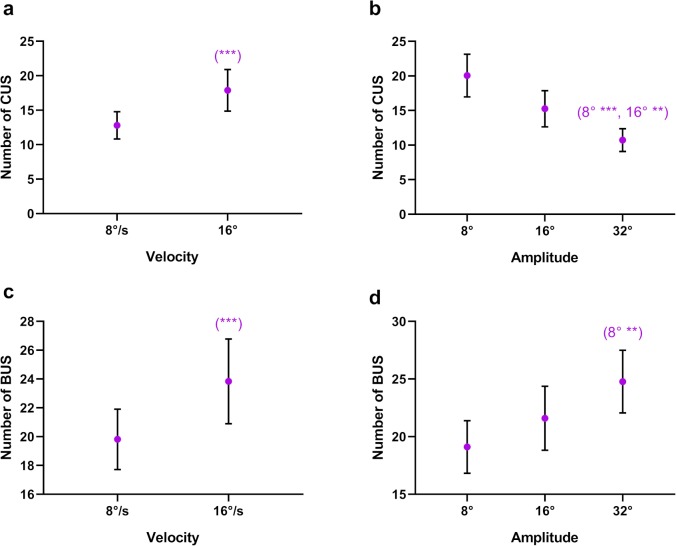
Mean number of catch-up saccades (CUS) as a function of target (a) velocity, (b) amplitude. Mean number of back-up saccades (BUS) as a function of target (a) velocity, (b) amplitude. The significance of post hoc comparisons is displayed above the data. The format and depiction of the levels of statistical significance are the same as in Figure 4.

We further examined these saccades by considering whether they were catch-up or back-up saccades. We defined catch-up and back-up saccades as those saccades made towards and away from the target position, respectively. There was a significant main effect of velocity on the number of catch-up (*F*_1,14_ = 22.730, *P* < 0.001, *η*^2^ = 0.619) and back-up (*F*_1,14_ = 21.789, *P* < 0.001, *η*^2^ = 0.609) saccades, with more of each type of saccade initiated at the higher target velocity (Figs. 9a, 9c). Similarly, there was a main effect of amplitude on catch-up (*F*_2,28_ = 19.583, *P* < 0.001, *η*^2^ = 0.583) and back-up (*F*_2,28_ = 6.177, *P* = 0.006, *η*^2^ = 0.306) saccades. More catch-up saccades were initiated at the smaller amplitude (Fig. 9b), whereas more back-up saccades were initiated at the larger amplitude (Fig. 9d).

#### Saccade Amplitude

**Figure 10:**
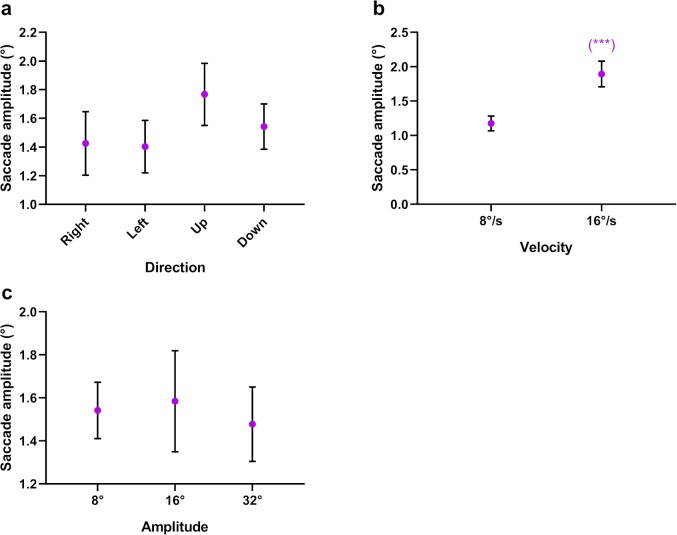
Mean saccade amplitude as a function of target (a) direction, (b) velocity, and (c) amplitude. The significance of post hoc comparisons is displayed above the data. The format and depiction of the levels of statistical significance are the same as in Figure 4.

We collectively analyzed the amplitudes of all saccades, whether catch-up or back-up. There was a significant main effect of velocity on saccade amplitude (*F*_1,14_ = 46.306, *P* < 0.001, *η*^2^ = 0.768), with larger amplitude saccades initiated at the higher target velocity (Fig. 10b). There was no significant main effect for either direction (*F*_1.71,24.00_ = 5.391, *P* = 0.051, *η*^2^ = 0.278) or amplitude (*F*_2,2_) = 0.576, *P* = 0.568, *η*^2^ = 0.040) on saccade amplitude.

#### Saccades and Accuracy of Pursuit

**Figure 11:**
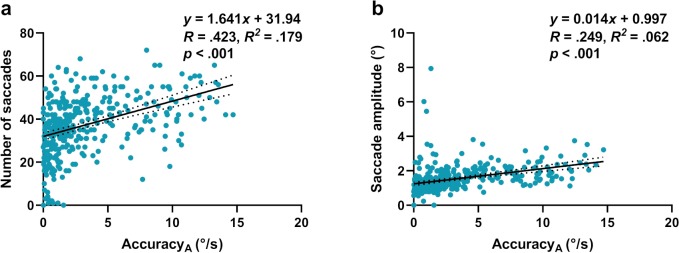
Correlation plots for (a) number of saccades and (b) saccade amplitude against pursuit accuracy along the target trajectory. Each data point represents the results of one trial combination (e.g., rightward pursuit at 8°/s × 32°) for one participant The *solid black trend line* represents the regression equation and the *dashed line* indicates the corresponding 95% confidence intervals.

We hypothesized that, as the accuracy of eye velocity along the target trajectory decreased, a greater number of saccades would be made. Furthermore, these saccades would have a larger amplitude to reduce the position and velocity error between the eye and target. We found significant regression equations for saccade number and amplitude (both *P* < 0.001), and the correlation coefficients indicated that eye velocity accuracy along the target trajectory was a moderate predictor of the number of saccades (*r* = 0.432; Fig. 11a) and a weak predictor of saccade amplitude (*r* = 0.249; Fig. 11b).

### Fixation

We compared fixation data against a pursuit condition that was known to yield high performance, in this case an 8°/s target moving rightward across 32°. We compared the magnitude of the precision along the major and minor axes for conditions of fixation and pursuit. However, as the fixation target was stationary (i.e., 0°/s), it was obviously not possible to compare accuracy along and orthogonal to a trajectory by using the *x* and *y* coordinates of the respective isocontour centroids. Instead, we compared the magnitude of the vector from the origin of the eye velocity distribution (Fig. 1b, black dot representing graph origin); that is, the target, to the isocontour centroid (Fig. 1b, blue dot representing isocontour center).

**Figure 12:**
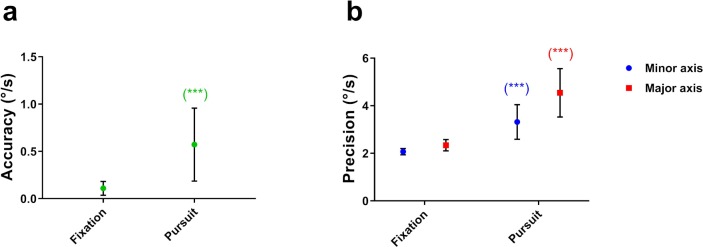
(a) Mean accuracy as a function of eye movement type. (b) Mean precision as a function of eye movement type by velocity isocontour axis. The significance of post hoc comparisons is displayed above the data. The format and depiction of the levels of statistical significance are the same as in Figure 4.

**Figure 13:**
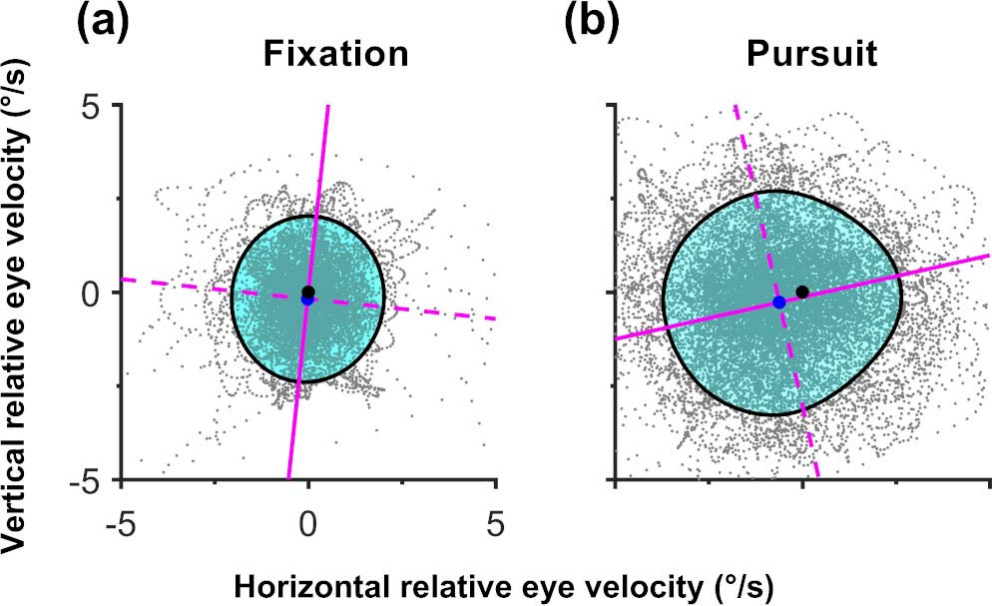
A summary of the differences between fixation and rightward pursuit (8°/s across 32°) using the data for participant 15. The format of each isocontour figure is the same as in Figure 1.

Compared to the pursuit condition, our results showed that eye velocity during fixation (i.e., fixational eye movements) was more accurate (*t*[14] = −5.04, *P* < 0.001) and more precise along both the minor (*t*[14] = 4.00, *P* < 0.001) and major (*t*[14] = 5.17, *P* < 0.001) axes (Fig. 12). A summary of the differences in the two classes of eye movements for a single typical participant is shown in Figure 13. It is evident that the overall precision is greater for fixation than in pursuit and that there is more variability in the vertical than horizontal axes. These results are largely in agreement with those reported by Cherici et al.^13^

## Discussion

The principal motivation behind this study was to explore the shortcomings of pursuit gain as an ubiquitous, and indeed almost exclusive, measure of pursuit performance, in typical and atypical populations. Existing measurements of pursuit performance relate only to accuracy rather than precision, and do not consider eye velocities other than those along the target heading. This is in marked contrast to analyses of other eye movements that do take account of all directions of movement. Consequently, pursuit gain only reveals a small facet of actual pursuit performance. Furthermore, the use of pursuit gain assumes that eye velocity orthogonal to the target trajectory is accurate and precise.

In the current study, we applied those methods commonly associated with characterizing the 2D accuracy and precision of eye position during fixation^13^ to eye velocity during pursuit. We also considered how each of our three experimental manipulations of pursuit direction, velocity, and amplitude impacted performance. In doing so, we demonstrated that the retinal image velocity is in a state of flux in directions other than the pursuit target, just as in fixation.

By examining the orientation of the major axes of eye velocity isocontours with respect to the target trajectory, we confirmed our prediction that the precision of eye velocity should be lower along the target trajectory than orthogonal to it. Early studies of smooth pursuit claimed that eye velocity imprecision along the target trajectory did not exceed the magnitude of fixational micro-drifts;^15^ however, in this study, we found the precision along the target trajectory far exceeds this, in agreement with others.^11^ Indeed, when we compared the precision of the minor axes of velocity isocontours for fixation and pursuit, those for pursuit were significantly less precise. In other words, the variability in eye velocity orthogonal to the target trajectory during pursuit is likely to exceed even fixational micro-drifts. The asymmetry of precision along and orthogonal to the target trajectory may reflect velocity ‘ringing,' where eye velocity during steady state pursuit oscillates about a particular value.^29^

We found that, during horizontal pursuit, the mean orientation of the major axis of the velocity isocontour also was horizontal, but that there was considerable individual variation of the individual major axis orientations. This would seem to be due to a more symmetric distribution of imprecision between the two axes; that is, a higher shape factor. This would have resulted in a less pronounced major axis. In this case, a small shift in the distribution of imprecision between the two axes would impact considerably on the major axis orientation.

We had predicted that eye velocities orthogonal to the target trajectory should be more precise during vertical than horizontal pursuit. While the shape factor of the eye velocity isocontours seemed to indicate this was the case, we found no significant differences in the precision orthogonal to the target trajectory with direction. However, changes in shape factor are ambiguous; it is not possible to determine which axis, or axes, are responsible for any change. In parallel, we chose to measure the precision along the minor and major axes but observed no significant differences in either with pursuit direction. The greater asymmetry of precision distribution during vertical pursuit could be attributed, in part, to a higher precision orthogonal to the target trajectory (i.e., a smaller minor axis) when compared to that during horizontal pursuit. In other words, vertical eye velocities during horizontal pursuit are subject to a more variable error than horizontal eye velocities during vertical pursuit. This variable error may reflect underlying differences in the arrangement of the extraocular muscles. Unlike horizontal eye position and velocity, no single pair of extraocular muscles controls vertical eye position and velocity. Instead, two pairs of extraocular muscles are involved, and as horizontal gaze angle varies, the relative contribution from each extraocular muscle pair varies.

Our study did not fully confirm our final prediction. We found that target amplitude did not affect eye velocities orthogonal to the target trajectory, whereas target velocity did. This observation may be related to lower pursuit accuracy orthogonal to the target trajectory as the target velocity increased. A lower accuracy would suggest that the target was not being continuously foveated. Indeed, this would be consistent with the results of Shanidze et al.,^14^ who found that small targets are not always foveated during pursuit. In typical observers, such eccentrically viewed targets have been shown to increase the variability of fixation eye movements,^30^ and may have had a similar impact on pursuit eye velocity.

We noted that, as target amplitude decreased, pursuit became less accurate and less precise. If the peak-to-peak amplitude of the target was further decreased to the point where the target was effectively stationary, our results would predict that, under these circumstances, eye velocity control would be worst. However, comparing the results of pursuit to those of fixation showed that the accuracy and precision during fixation was superior to even the most optimal of pursuit conditions. This observation suggested that the mere act of following a moving target reduces accuracy and precision when compared to fixation. We hypothesize that this finding is related to the frequency of the pursuit target oscillation. Modulating either the target amplitude with a fixed speed or modulating speed with a fixed amplitude will alter the target frequency. Several studies^31^–^34^ have shown that increasing target frequency results in lower pursuit gain, so we hypothesized that the smaller amplitude in our experimental protocol lowered pursuit performance because this resulted in an increased frequency.

The results for our subsidiary fixation experiment were largely in agreement with the polar plots of eye velocity during fixation reported by Cherici et al.^13^ While our precision values were typically larger (2.06 ± 0.24°/s minor axis; 2.34 ± 0.43°/s major axis), this was most likely due to methodologic differences, such as the lack of a bite bar, a larger fixation stimulus, longer fixation duration, and/or a lack of a second-stage, manual calibration.

For horizontal and vertical pursuits, we found eye velocities orthogonal to the target trajectory were inaccurate and imprecise. Velocity mismatch between the eye and target has been widely accepted as the stimulus for smooth pursuit. Thus, imprecision of eye velocity should serve as a stimulus for pursuit. However, to what extent this previously undocumented ongoing retinal image motion actually serves as a stimulus for driving pursuit remains to be determined. Nonetheless, we would argue that it should be considered in future models of pursuit.

Our analytical approach replicated several other pursuit-related findings for eye velocity along the target trajectory and extended these data. For example, we found a horizontal–vertical accuracy anisotropy:^34^–^37^ horizontal pursuit is more accurate than vertical pursuit. No differences were found between leftward and rightward pursuit accuracy, or between downward and upward target pursuit accuracy. A vertical asymmetry in pursuit accuracy has been reported previously.^35^ However, our data (Table 2) agree with others who have attributed this to idiosyncratic differences in participant performance.^36^ We also found that eye velocity was less accurate with increased target velocity^11^,^34^,^35^,^37^ and that accuracy decreases with smaller target amplitudes.^34^,^38^ Finally, we observed that precision decreased with increased target velocity.^11^

**Table 2:** The Percentage of Trial Combinations Where Accuracy Along the Target Trajectory was Greater for Downward Pursuit Than for Upward Pursuit

Observer	% of Trial Combinations
p01	50.00
p02	83.33
p03	100.00
p04	0.00
p05	50.00
p06	16.67
p07	66.67
p08	50.00
p09	83.33
p10	0.00
p11	0.00
p12	50.00
p13	66.67
p14	33.33
p15	66.67

These data suggest idiosyncratic differences in vertical pursuit accuracy rather than an up–down asymmetry.

Our method of saccade selection and removal allowed for the possibility that some saccades were not removed from the pursuit data and were included in the analyzed velocity isocontours. However, based on our method of saccade selection (mean eye velocity plus a multiple of the SD), any undetected saccades would be of low peak velocity and, hence, short duration.^39^ In addition, by selecting the 68% velocity isocontour for analysis, we reduced the potential for eye velocity outliers. Thus, of the many thousands of genuine pursuit samples, the presence of any undetected saccades in the underlying velocity distributions would be unlikely to distort the resulting velocity isocontour.

We found that there was an increase in the number of saccades initiated during pursuit at the higher target velocity and the smallest pursuit amplitude. That these trial parameters resulted in the lowest accuracy along the target trajectory indicated that the saccades were used to compensate for the velocity error. However, given that accuracy along the target trajectory was a poor predictor for the number and amplitude of saccades, this suggested that the primary role of saccades during pursuit is to reduce the position error, rather than the velocity error, consistent with the previous report of de Brouwer et al.^28^ Our saccade data were in broad agreement with the results of a recent study of small amplitude pursuit in monkeys.^34^ These investigators found that, at a fixed target amplitude (0.5°), increasing the target velocity (i.e., frequency) resulted in an increase in the number (and amplitude) of catch-up saccades, although the number of catch-up saccades fell off at target frequencies greater than 1 Hz. However, while we found the number of catch-up saccades decreased with increased amplitude, these investigators also found that at a fixed target frequency (0.5 Hz; i.e., fixed velocity), the number (and amplitude) of catch-up saccades increased with target amplitude. This discrepancy may reflect the much larger pursuit amplitudes (i.e., >2°) used in our current study, or because we have not maintained a fixed target frequency as the target amplitude varied.

In our analyses, we restricted the pursuit data to the central 70% of each sweep to obtain a representative estimate of steady state pursuit. As such, we remained confident that our measures are representative of typical observers. However, it is worth noting that the temporal relationship between eye movement samples was not retained in our analytical approach, and so it cannot be ascertained whether accuracy and precision varies during pursuit.

Our study involved pursuit under simplified conditions in which the target moved at constant velocity with periodic oscillations, making it highly predictable.^40^–^42^ The extent to which pursuit was foveally driven in our observers, and how our findings would differ under more complicated target motion, such as a random walk of sinusoids,^43^ remains unclear. Nonetheless, our results should be considered the upper limits for pursuit performance in typical observers.

It is well recognized that too little or too much retinal image motion impedes visual perception. Our results raised the question of whether the 2D pattern of retinal image motion during pursuit observed is visually detrimental. For example, one study has shown that detection thresholds for horizontally oriented luminance gratings were lower during horizontal pursuit than fixation.^44^

Our more comprehensive analysis is undoubtedly more informative than gain alone, and so may be more effective in detecting subtle deviations from normal pursuit behavior within atypical populations. Indeed, the utility of our 2D analysis of eye velocity is not limited to pursuit. We anticipate that it will facilitate a more detailed investigation and modeling of other types of slow eye movements that are currently quantified only using gain, including optokinetic nystagmus and vestibulo-ocular response. We would certainly argue that, whatever eye movement is investigated and whether in typical or atypical populations, outcome measures should include precision alongside accuracy, and wherever possible, eye velocity should be quantified two-dimensionally. This is likely to be particularly important when assessing slow phase eye movements in those with neurologic disorders, such as Parkinson's disease, Huntington's disease, and others to facilitate the identification of an early onset of abnormalities, which could then serve as useful biomarkers for assessing disease progression and/or the efficacy of treatments in a given individual.
